# Effects of Peptidoglycan, Lipoteichoic Acid and Lipopolysaccharide on Inflammation, Proliferation and Milk Fat Synthesis in Bovine Mammary Epithelial Cells

**DOI:** 10.3390/toxins12080497

**Published:** 2020-08-02

**Authors:** Yongjiang Wu, Yawang Sun, Zhu Zhang, Juncai Chen, Guozhong Dong

**Affiliations:** College of Animal Science and Technology, Southwest University, Beibei District, Chongqing 400716, China; wuyongjiang@email.swu.edu.cn (Y.W.); syaw507@swu.edu.cn (Y.S.); b20020901102@swu.edu.cn (Z.Z.); juncaichen@swu.edu.cn (J.C.)

**Keywords:** peptidoglycan, lipoteichoic acid, lipopolysaccharide, inflammation, proliferation, milk fat, bovine mammary epithelial cells

## Abstract

The mammary gland of the cow is particularly susceptible to infections of a wide range of pathogenic bacteria, including both Gram-positive and Gram-negative bacteria. The endotoxins of these pathogenic bacteria include peptidoglycan (PGN), lipoteichoic acid (LTA) and lipopolysaccharide (LPS), and they are the pathogen-associated molecular patterns (PAMPs) to induce mastitis. LPS can directly inhibit proliferation and milk fat synthesis of bovine mammary epithelial cells (BMECs) while inducing mastitis, but it is unclear whether PGN and LTA also have such effects. Furthermore, since the three PAMPs usually appear simultaneously in the udder of cows with mastitis, their synergistic effects on proliferation and milk fat synthesis of BMECs are worth investigating. The immortalized BMECs (MAC-T cells) were stimulated for 24 h using various concentrations of PGN, LTA and LPS, respectively, to determine the doses that could effectively cause inflammatory responses. Next, the cells were stimulated for 24 h with no endotoxins (CON), PGN, LTA, LPS, PGN + LTA, and PGN + LTA + LPS, respectively, with the predetermined doses to analyze their effects on proliferation and milk fat synthesis of BMECs. PGN, LTA and LPS successfully induced inflammatory responses of BMECs with doses of 30, 30 and 0.1 μg/mL, respectively. Although the proliferation of BMECs was significantly inhibited in the following order: LTA < PGN + LTA < PGN + LTA + LPS, there was no change in cell morphology and cell death. LTA significantly promoted the expression of fatty acid synthesis-related genes but did not change the content of intracellular triglyceride (TG), compared with the CON group. The mRNA expression of fatty acid synthesis-related genes in the LPS group was the lowest among all the groups. Meanwhile, LPS significantly decreased the content of intracellular non-esterified fatty acids (NEFAs) and TG, compared with the CON group. PGN had no effects on milk fat synthesis. Co-stimulation with PGN, LTA and LPS significantly increased the expression of fat acid synthesis-related genes and the intracellular NEFAs, but decreased intracellular TG, compared with sole LPS stimulation. Collectively, PGN, LTA and LPS showed an additive effect on inhibiting proliferation of BMECs. The promoting role of LTA in fatty acid synthesis might offset the negative effects of LPS in this regard, but co-stimulation with PGN, LTA and LPS significantly decreased intracellular TG content.

## 1. Introduction 

Mastitis is one of the most prevalent and devastating inflammatory diseases in dairy cows over the world. It results in severe economic losses to the dairy industry, due to reduced milk yield and quality as well as increased treatment costs and the cull rate of cows. The annual total economic cost of mastitis was estimated at approximately 2 billion dollars and 1.55 billion euros in the USA and Europe, respectively [1,2].

When a cow suffers from mastitis, its mammary epithelial cells can synthesize and secrete a large number of proinflammatory factors, mainly including interleukin (IL)-1β, IL-6, IL-8, tumor necrosis factor (TNF)-α and other cytokines. This might disturb the proliferation and milk synthesis of bovine mammary epithelial cells (BMECs), resulting in a decrease in milk yield and quality. The amounts and secretory activity of BMECs are related to milk yield [3], and the lipid content of milk, is an important indicator of milk quality [4,5]. Triglyceride (TG) accounted for more than 98% of milk lipid [6]. Fatty acids can be rapidly taken up and converted into lipid droplets by the lactating mammary gland [7]. Non-esterified fatty acid (NEFA) is a source of fatty acids and can increase milk fat synthesis [8]. A variety of genes, such as fatty acid synthase (*FASN*), acetyl coenzyme-A carboxylase 1 (*ACACA*), and stearoyl-CoA desaturase (*SCD*), etc., are involved in milk fat synthesis [9,10,11,12,13,14]. FASN catalyzes the synthesis of long-chain fatty acids [15], ACACA is the rate-limiting enzyme catalyzing the first reaction step of fatty acid synthesis [16], and SCD is responsible for catalyzing the synthesis of mono-saturated fatty acids [17].

In practical production, the udder of cows is particularly susceptible to infections of a variety of pathogenic bacteria, including both Gram-positive and Gram-negative bacteria [18,19,20]. *S. aureus* and *E. coli* are the most common Gram-positive and Gram-negative bacteria, respectively, which induce mastitis [21,22]. Their cell wall components (endotoxins), including peptidoglycan (PGN), lipoteichoic acid (LTA) and lipopolysaccharide (LPS), are released during the process of proliferation or/and after death. Many previous studies reported that LPS, a pathogen-associated molecular pattern (PAMP), induced inflammatory responses of BMECs, and it could also simultaneously inhibit the proliferation of BMECs [23,24,25,26] and decrease milk fat synthesis in BMECs by suppressing the expression of fat acid synthesis-related genes [4,27,28]. PGN and LTA are also PAMPs that cause inflammatory responses and affect lactation in the mammary gland of cows [29,30]. However, there are few studies reporting the effects of PGN and LTA on the proliferation and milk fat synthesis of BMECs. Meanwhile, LPS, PGN and LTA are usually present simultaneously in the udder of cows with mastitis, and it is currently unclear whether PGN, LTA and LPS display a synergistic effect on proliferation and milk fat synthesis of BMECs.

Thus, this present work is aimed to compare the different effects of PGN, LTA and LPS on the proliferation and milk fat synthesis of BMECs and to explore whether there exists a synergistic effect for them to adversely affect the proliferation and milk fat synthesis in BMECs. 

## 2. Results

### 2.1. The Effects of Various Concentrations of PGN, LTA and LPS on Gene Expression of Cytokines in BMECs

**Figure 1:**
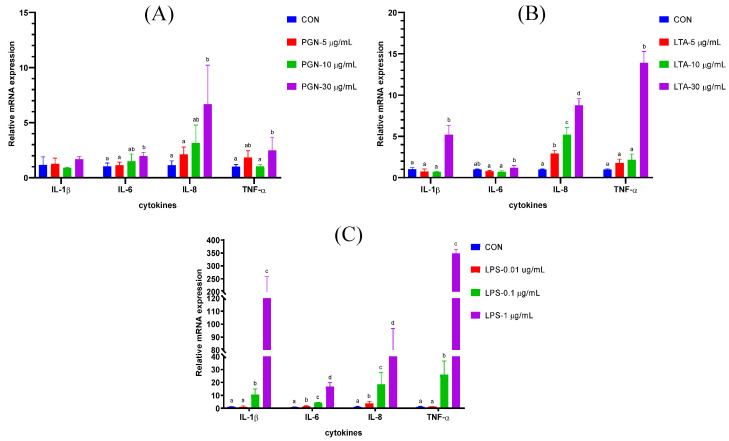
The effects of various concentrations of peptidoglycan (PGN), lipoteichoic acid (LTA), and lipopolysaccharide (LPS) on the gene expression of cytokines in bovine mammary epithelial cells (BMECs). (**A**) The effects of various concentrations peptidoglycan (PGN) on the gene expression of cytokines. (**B**) The effects of various concentrations lipoteichoic acid (LTA) on the gene expression of cytokines. (**C**) The effects of various concentrations lipopolysaccharide (LPS) on the gene expression of cytokines. The BMECs were stimulated for 24 h with 0, 5, 10, and 30 μg/mL PGN; with 0, 5, 10, and 30 μg/mL LTA; and with 0, 0.01, 0.1, and 1 μg/mL LPS; respectively. After stimulation, the cells were collected, and then total RNA was isolated for reverse transcription quantitative real-time polymerase chain reaction analyses. Data were analyzed with one-way analysis of variance followed by Duncan’s multiple comparison test and presented as mean ± standard deviation. Different lowercase letters (a, b, c, d) on the top of bars indicate significant differences (n = 3, *p* < 0.05) among the groups. *IL-1β*, interleukin-1β; *IL-6*, interleukin-6; *IL-8*, interleukin-8; *TNF-α*, tumor necrosis factor-α; CON, control group.

As shown in Figure 1A, compared with the CON group, only 30 μg/mL PGN significantly increased the mRNA expression of cytokines, including *IL-6*, *IL-8* and *TNF-α*. The effects of PGN on *IL-8* were the strongest among the four cytokines. As shown in Figure 1B, compared with the CON group, 30 μg/mL LTA significantly increased the mRNA expression of cytokines, including *IL-1β*, *IL-8* and *TNF-α*. The mRNA expression of *IL-6* in the 30 μg/mL LTA group was significantly higher than that in the 5 μg/mL LTA and 10 μg/mL LTA groups. The mRNA expression of *IL-8* significantly increased with increasing concentrations of LTA. As shown in Figure 1C, compared with the CON group, 0.01 μg/mL LPS only significantly increased the mRNA expression of *IL-6* and *IL-8*. When the concentration of LPS reached 0.1 μg/mL, the mRNA expression of all the four cytokines, including *IL-1β*, *IL-6*, *IL-8* and *TNF-α* significantly increased, compared with the CON group and the 0.01 μg/mL LPS group. Moreover, the mRNA expression of all the four cytokines in the 1 μg/mL LPS group was significantly higher than other groups.

### 2.2. The Effects of PGN, LTA and LPS on Proliferation of BMECs

**Figure 2:**
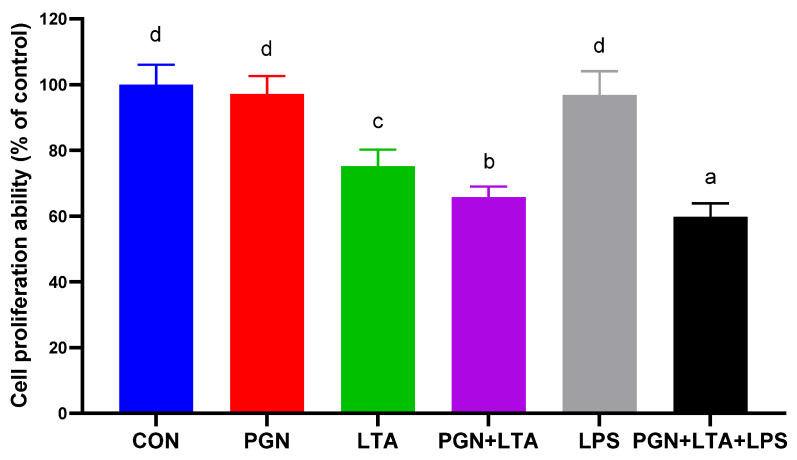
The effects of PGN, LTA and LPS on proliferation of bovine mammary epithelial cells (BMECs). After stimulation for 24 h with no endotoxins (CON), 30 μg/mL PGN, 30 μg/mL LTA, 0.1 μg/mL LPS, 30 μg/mL PGN + 30 μg/mL LTA, or 30 μg/mL PGN + 30 μg/mL LTA + 0.1 μg/mL LPS, 10 μL of Cell Counting Kit-8 (CCK-8) solution was added to each treatment well and incubated for 4 h. Next, the optical density value (absorbance) was measured at 450 nm and normalized with that of the control group. Data were analyzed with one-way analysis of variance followed by Duncan’s multiple comparison test and presented as mean ± standard deviation. Different lowercase letters (a, b, c, d) on the top of bars indicate significant differences (n = 10, *p* < 0.05) among the groups. CON, control group; PGN, peptidoglycan group; LTA, lipoteichoic acid group; LPS, lipopolysaccharide group; PGN + LTA, the group of co-stimulation with PGN and LTA; PGN + LTA + LPS, the group of co-stimulation with PGN, LTA and LPS.

As shown in Figure 2, compared with the CON group, PGN and LPS had no significant effects on proliferation of BMECs, but LTA significantly inhibited proliferation of BMECs. Co-stimulation with PGN and LTA further inhibited proliferation of BMECs, compared with sole LTA stimulation. In addition, co-stimulation with PGN, LTA and LPS greatly inhibited proliferation of BMECs, compared with the co-stimulation with PGN and LTA.

**Figure 3:**
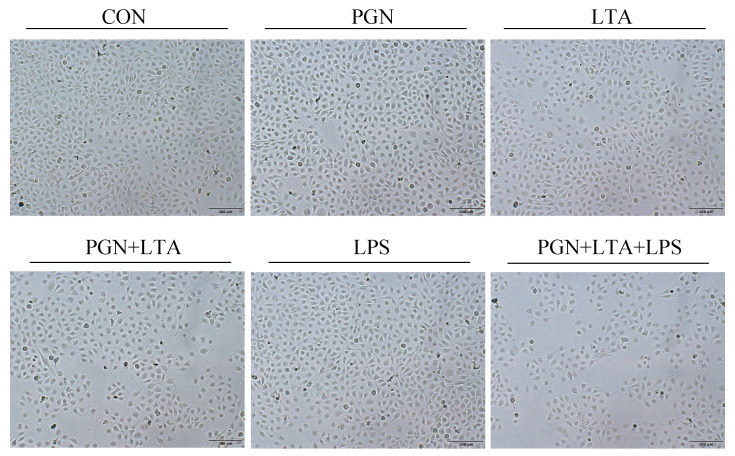
The cell morphology observed after stimulation with PGN, LTA and LPS. After stimulation for 24 h with no endotoxins (CON), 30 μg/mL PGN, 30 μg/mL LTA, 0.1 μg/mL LPS, 30 μg/mL PGN + 30 μg/mL LTA, or 30 μg/mL PGN + 30 μg/mL LTA + 0.1 μg/mL LPS, the cell morphology was observed and photographed with an inverted microscope (×100). CON, control group; PGN, peptidoglycan group; LTA, lipoteichoic acid group; LPS, lipopolysaccharide group; PGN + LTA, the group of co-stimulation with PGN and LTA; PGN + LTA + LPS, the group of co-stimulation with PGN, LTA and LPS.

As shown in Figure 3, the cells in all groups had a cobblestone-like shape, and their cell morphology was not different. In addition, there were not a large number of floating dead cells in the media of all the groups. The number of cells in the group of co-stimulation with PGN, LTA and LPS was the lowest among all the groups, which further confirmed the proliferation results, indicating that the co-stimulation might inhibit cell growth, but did not cause cell death. 

### 2.3. The Effects of PGN, LTA and LPS on Milk Fat Synthesis

#### 2.3.1. The Effects of PGN, LTA and LPS on the Expression of Fat Acid Synthesis-Related Genes

**Figure 4:**
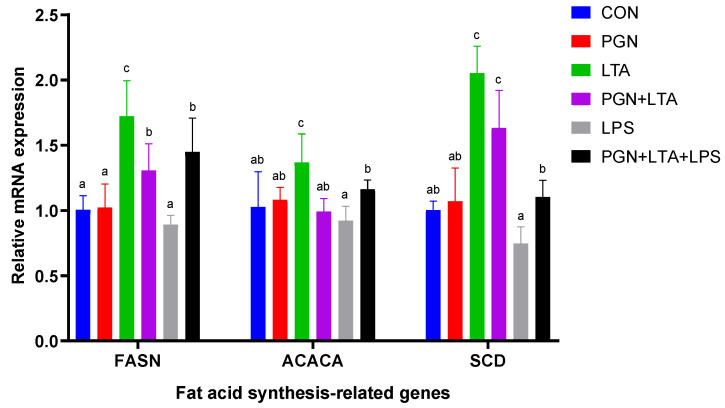
The effects of PGN, LTA and LPS on the expression of fat acid synthesis-related genes. After stimulation for 24 h with no endotoxins (CON), 30 μg/mL PGN, 30 μg/mL LTA, 0.1 μg/mL LPS, 30 μg/mL PGN + 30 μg/mL LTA, or 30 μg/mL PGN + 30 μg/mL LTA + 0.1 μg/mL LPS, the cells were collected, and then total RNA was isolated for reverse transcription quantitative real-time polymerase chain reaction analyses. Data were analyzed with one-way analysis of variance followed by Duncan’s multiple comparison test and presented as mean ± standard deviation. Different lowercase letters (a, b, c) on the top of bars indicate significant differences (n = 6, *p* < 0.05) among the groups. *FASN*, fatty acid synthase; *ACACA*, acetyl coenzyme-A carboxylase 1; *SCD*, stearoyl-CoA desaturase; CON, control group; PGN, peptidoglycan group; LTA, lipoteichoic acid group; LPS, lipopolysaccharide group; PGN + LTA, the group of co-stimulation with PGN and LTA; PGN + LTA + LPS, the group of co-stimulation with PGN, LTA and LPS.

As shown in Figure 4, the mRNA expression of fat acid synthesis-related genes, including *FASN*, *ACACA* and *SCD* in the LTA group, was significantly higher than other groups. The mRNA expression of fat acid synthesis-related genes in the LPS group was, overall, the lowest among all the groups. Compared with the CON group, the PGN had no significant effects on mRNA expression of all the fat acid synthesis-related genes. Compared with sole LPS stimulation, co-stimulation with PGN, LTA and LPS significantly promoted the expression of all the fat acid synthesis-related genes, and co-stimulation with PGN and LTA also significantly promoted the expression of *FASN* and *SCD*.

#### 2.3.2. The Effects of PGN, LTA and LPS on the Content of Intracellular NEFAs and TG 

**Figure 5:**
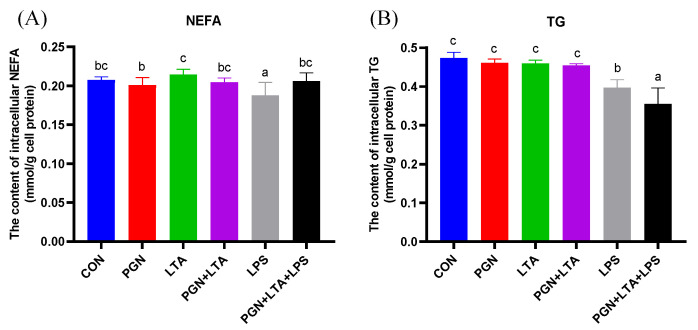
The effects of PGN, LTA and LPS on the content of intracellular non-esterified fatty acids (NEFAs) and triglyceride (TG). (**A**) The content of intracellular NEFAs. (**B**) The content of intracellular TG. After stimulation for 24 h with no endotoxins (CON), 30 μg/mL PGN, 30 μg/mL LTA, 0.1 μg/mL LPS, 30 μg/mL PGN + 30 μg/mL LTA, or 30 μg/mL PGN + 30 μg/mL LTA + 0.1 μg/mL LPS, the cells were collected and then broken for measuring intracellular NEFAs and TG by the corresponding kits. Data were analyzed with one-way analysis of variance followed by Duncan’s multiple comparison test and presented as mean ± standard deviation. Different lowercase letters (a, b, c) on the top of bars indicate significant differences (n = 6, *p* < 0.05) among the groups. CON, control group; PGN, peptidoglycan group; LTA, lipoteichoic acid group; LPS, lipopolysaccharide group; PGN + LTA, the group of co-stimulation with PGN and LTA; PGN + LTA + LPS, the group of co-stimulation with PGN, LTA and LPS.

As shown in Figure 5, compared with the CON group, only the LPS significantly decreased the content of intracellular NEFAs. The content of intracellular NEFAs in the LTA group was the highest among all the groups and significantly higher than that in the PGN and LPS groups. There was no significant difference for TG among the CON, PGN, LTA, and PGN + LTA groups. The content of intracellular TG in the LPS group was significantly lower than that in the CON group, but significantly higher than that in the group co-stimulated with PGN, LTA and LPS.

## 3. Discussion

PGN, LTA and LPS at different doses often induce different degree of inflammatory responses in BMECs. In a study by Yu et al. [31], they examined the effects of various concentrations (1 ng/mL to 10 μg/mL) of PGN, LTA and LPS on gene expression of *IL-8* in BMECs, and found that, compared with the control, all the concentrations of PGN did not significantly increase the gene expression of *IL-8*; LTA and LPS significantly increased the gene expression of *IL-8*, when the concentration reached 10 μg/mL and 1 μg/mL, respectively. LPS at a dose of 10 μg/mL significantly increased the gene expression of *IL-8*, compared with 1 μg/mL of LPS [31]. These results were generally consistent with our findings in the present study. In addition, we further analyzed the effects of various concentrations of PGN, LTA and LPS on other cytokines, including *IL-1β*, *IL-6* and *TNF-α*, and found that 30 μg/mL PGN or LTA leaded to an overall increase in gene expression of the cytokines in BMECs. In our previous study, we found that sole 30 μg/mL PGN or LTA stimulation also increased the expression of cytokines in BMECs, and co-stimulation with PGN and LTA displayed an additive effect on the expression of cytokines [30]. Similar to our present study, a study showed that co-treatment with 30 μg/mL PGN and LTA up-regulated nuclear factor-κB (NF-κB) activity and the expression of various inflammation-related genes in BMECs [32]. These results indicated that 30 μg/mL PGN or LTA could successfully induce inflammatory responses by overall enhancing the expression of cytokines in BMECs. 

LPS usually was used to construct an inflammatory model. The construction of mastitis model of cows was frequently based on 1 μg/mL LPS stimulation for BMECs in vitro in many studies [33,34,35,36,37]. There were some studies which reported that a lower concentration of LPS, such as 0.1 [38,39], 0.2 [40,41], 0.5 [42] μg/mL and so on, could also induce inflammatory responses of BMECs. In the present study, 0.01 μg/mL LPS only significantly promoted the gene expression of *IL-6* and *IL-8*. The expression of cytokines displayed an overall increase, when LPS concentration was raised to 0.1 μg/mL. In our previous study, 0.1 μg/mL LPS induced an overall increase in the expression of cytokines [43] and caused hypomethylation of genes involved in the immune pathways [44], which successfully promoted inflammatory responses of BMECs. In a study by Guenther et al. [38], they found that 0.1 μg/mL LPS enhanced the expression of inflammatory factors, including *IL-8* and C-C motif chemokine (*CCL*) *5*. In the present study, the expression of all the inflammatory factors induced by 1μg/mL LPS was significantly higher than that by 0.1 μg/mL LPS. Thus, we suggest that the severe mastitis model can be constructed using 1μg/mL LPS, and the construction of the mild mastitis model can be based on 0.1 μg/mL LPS. 

In this study, sole PGN or LPS stimulation had no significant effects on proliferation of BMECs, but sole LTA stimulation significantly inhibited the proliferation. Several previous studies have shown that PGN promoted the proliferation of human T-cell [45], mesenchymal stem cells [46], malignant glioma cells [47], and rat fibroblasts [48]. However, a study found that PGN did not affect the proliferation of rat fibroblasts [49]. The difference in the results of these studies might be due to differences in cell type and PGN dosage. Many previous studies suggested that LPS inhibited the proliferation of BMECs [23,24,25,26], but our results did not support the findings. Perhaps it is because high concentrations of LPS, such as 0.5 [23], 1 [23,24,25], 5 [23], 100 μg/mL [26] and more, were used in those studies, which caused very severe inflammation and blocked cell proliferation or growth in BMECs. Although a low concentration of LPS (0.1 μg/mL) used in the present study also induced a certain degree of inflammatory response, it was insufficient to inhibit proliferation of BMECs. The previous studies demonstrated that LTA inhibited cell proliferation [50,51,52], which was consistent with the results in the present study. Meanwhile, in this study, co-stimulation with PGN and LTA inhibited proliferation of BMECs to a greater degree than sole PGN or LTA stimulation. In our previous study, the differentially expressed genes (DEGs) induced by co-stimulation with PGN and LTA were significantly enriched in the cell cycle pathway [30], which indicated that co-stimulation with PGN and LTA might inhibited proliferation of BMECs by regulating cell cycle. In addition, co-stimulation with PGN, LTA and LPS further inhibited proliferation of BMECs than co-stimulation with PGN and LTA. Normal BMECs have a typical cobblestone-like shape and clear boundaries [53]. In the present study, the cells in all the groups also presented a cobblestone-like shape and no change in cell morphology and cell death. These results indicated that PGN, LTA and LPS had a synergistic effect on inhibiting proliferation of BMECs, but did not affect cell morphology and cell death. 

When BMECs were stimulated by PGN, LTA and LPS, the normal function of milk fat synthesis was also influenced. In a recent study by Jin et al. [54], the liver LO2 cell line cells and primary hepatocytes were stimulated for 16 h using 40 μg/mL PGN, which significantly increased their TG content and the expression of lipogenesis related genes, such as peroxisome proliferator-activated receptor (*PPAR*)-*γ*, sterol regulatory element binding protein (*SREBP*)*1*, *FASN*, etc. In the present work, the BMECs were incubated for 24 h with 30 μg/mL PGN, which only induced a certain level of inflammatory responses, but without any effects on milk fat synthesis. This was probably because of differences in cell treatment conditions or cell type. In our previous study [30], we found that the DEGs induced by LTA mainly participated in lipid metabolic process and significantly increased the expression of fat acid synthesis-related genes, including fatty acid binding protein (*FABP*)*3*, *SCD* and acyl-CoA synthetase long-chain family member (*ACSL*)*5* in BMECs. The expression up-regulation of fat acid synthesis-related genes could promote fatty acid biosynthesis and then increase the content of intracellular NEFAs and TG. In this study, LTA significantly promoted the expression of *FASN*, *ACACA* and *SCD*, and had the highest intracellular NEFA content among all the groups, which further confirmed our previous results [30]. However, LTA did not increase the content of intracellular TG. This is perhaps due to that the increased fatty acids were used for inflammatory responses [55,56], instead of being used for TG synthesis. In a study by Zebeli and Ametaj [57], they found that LPS caused stronger depression in milk fat content and declines in milk fat yield in lactating Holstein cows. He et al. [4] further explored the molecular mechanism of LPS inhibiting milk fat synthesis, and found that LPS down-regulated the expression of *FASN*, *ACACA*, acyl-CoA-binding protein (*ACBP*) and lipoprotein lipase (*LPL*) by suppressing PPAR-γ in BMECs. In addition, our previous research work also supported that LPS decreased the expression of *FASN*, *ACACA* and acyl-CoA synthetase short-chain family member (*ACSS*) 2 in BMECs [44,58]. In this study, the gene expression of *FASN*, *ACACA* and *SCD* in the LPS group were the lowest among all the groups. In addition, LPS significantly decreased the content of intracellular NEFAs and TG, compared with CON group. These results suggested that LPS inhibited milk fat synthesis by suppressing the expression of fat acid synthesis-related genes. Interestingly, LTA in combination with LPS and PGN significantly promoted the expression of fat acid synthesis-related genes and the content of intracellular NEFAs, compared with sole LPS stimulation, indicating that the promoting role of LTA in fatty acid synthesis offset the negative effects of LPS on fatty acid synthesis. However, co-stimulation with PGN, LTA and LPS significantly decreased the content of intracellular TG, compared with other groups, suggesting that the fatty acids might mainly participate in inflammatory responses, rather than in TG synthesis.

## 4. Conclusions

LTA (30 μg/mL) inhibited proliferation of BMECs, but PGN (30 μg/mL) and LPS (0.1 μg/mL) had no such effect. The three PAMPs have an additive effect on inhibiting proliferation of BMECs. LTA (30 μg/mL) promoted fatty acid synthesis, LPS (0.1 μg/mL) inhibited fatty acid synthesis and PGN (30 μg/mL) had no effect on fatty acid synthesis. Although LTA might offset the negative effects of LPS on fatty acid synthesis, co-stimulation with LTA, PGN and LPS decreased intracellular TG content. 

## 5. Materials and Methods

### 5.1. Cell Culture and Treatments

The bovine mammary epithelial cell line (MAC-T) was a generous gift from Professors Jianxin Liu and Hongyun Liu at the Institute for Dairy Research, Zhejiang University, China. The cell line, as a culture model of bovine mammary epithelial cells, has been frequently used in research, and its establishment and culture method were previously described in detail by Huynh et al. [59]. The cell suspension with more than 95% viable cells was initially plated into six-well plates (Corning, NY, USA) at a seeding density of 1 × 10^5^ cells per well in 2 mL of the same culture medium. The culture medium composition was the same as that in our previous study [30] and was changed every 24 h of incubation. All the cells in plates were incubated at 37 °C with 5% CO_2_ in an incubator. 

The culture medium separately containing PGN (*S. aureus*, Sigma-Aldrich, 77140, St. Louis, MO, USA) at 0, 5, 10 or 30 μg/mL, LTA (*S. aureus*, Sigma-Aldrich, L2515, St. Louis, MO, USA) at 0, 5, 10 or 30 μg/mL or LPS (*E. coli*, O111:B4, Sigma-Aldrich, L2630, St. Louis, MO, USA) at 0, 0.01, 0.1 or 1 μg/mL was prepared. When cells grew to 60%–70% confluence, they were stimulated for 24 h with the prepared culture medium to determine the dose that could successfully induce inflammatory responses of BMECs by reverse transcription quantitative real-time polymerase chain reaction (RT-qPCR) analyses. Next, cells were stimulated for 24 h separately using PGN, LTA, LPS, PGN + LTA and PGN + LTA + LPS with the screened doses to analyze their synergistic effects on proliferation and milk fat synthesis in BMECs.

### 5.2. Total RNA Isolation, Reverse Transcription, and RT-qPCR

After total cellular RNAs isolation using the TRIzol reagent (Invitrogen, Carlsbad, CA, USA), their purity and concentration were tested with a spectrophotometer (Implen, Munich, Germany). The obtained total RNA was used for reverse transcription to generate complementary DNA (cDNA). The iScript cDNA synthesis kit (Bio-Rad, Hercules, CA, USA) recommended the incubation program of reverse transcription as follows: 25 °C for 5 min, 46 °C for 20 min and 95 °C for 1 min. The obtained cDNA was used for RT-qPCR with the Ssofast EvaGreen Supermix kit (Bio-Rad, Hercules, CA, USA) in a BIO-RAD CFX Connect Real-Time System (Bio-Rad, Hercules, CA, USA). The cDNA (0.8 ug) used as the template was included in the 20 μL reaction system in each well of the 96-well PCR plates. Each cDNA sample was amplified 3 times using the primers of the target genes. The procedure of RT-qPCR amplification was as follows: 95 °C for 30 sec, 40 cycles at 95 °C for 5 sec, and 58 °C for 5 sec, and a melting curve analysis was added to the tail of the procedure to ensure specific amplification. The primers (Table S1) were designed with NCBI primer-BLAST (https://www.ncbi.nlm.nih.gov/tools/primer-blast/) and synthesized and purified by BGI Co., Ltd. (Shenzhen, China). The glyceraldehyde-3-phosphate dehydrogenase gene (*GAPDH*) was used as the internal reference gene for normalizing the expression data of the target genes. The classic 2^−ΔΔCT^ method was used to calculate relative mRNA expression of the target genes. 

### 5.3. Cell Proliferation Assay and Morphology Observation

Cell proliferation was measured by the Cell Counting Kit-8 (CCK-8) kit (Dojindo Molecular Technologies, Inc., Kumamoto, Japan). The cells were seeded into a 96-well plate at a density of 5 × 10^3^ cell per well containing 100 μL culture medium. At 24 h after stimulation with PGN, LTA, LPS, PGN + LTA or PGN + LTA + LPS, 10 μL of CCK-8 solution was added to each treatment well and incubated for 4 h. Then, the optical density value (absorbance) was measured at a wavelength of 450 nm by a microplate reader (Thermo Fisher Scientific, Waltham, MA, USA). All the absorbance data were normalized with those of the control group. The cell proliferation ability was expressed as a percentage. There were 10 biological replicates in each group.

The cell suspension with more than 95% viable cells was initially plated into six-well plates (Corning, NY, USA) at a seeding density of 1 × 10^5^ cells per well in 2 mL of the same culture medium. When the cells grown to about 60% confluence, they were stimulated for 24 h as described above. Next, the cell morphology was observed and photographed with an inverted microscope (Olympus, Tokyo, Japan).

### 5.4. Intracellular NEFAs and TG Detection

At 24 h after stimulation with no endotoxins (CON), PGN, LTA, LPS, PGN + LTA or PGN + LTA + LPS, the cells were collected and then broken up using an ultrasonic cell crusher (Sonics & Materials, Newtown, CT, USA). There were six biological replicates in each group. The protein concentrations of cell lysates were determined using the BCA Protein Assay Kit (Sangon Biotech, Shanghai, China). The intercellular TG and NEFAs were measured using the triglyceride assay kit and the non-esterified free fatty acids assay kit (Nanjing Jiancheng Bioengineering Institute, Nanjing, China), respectively, according to the manufacturer’s instructions. The absorbance values were read on a microplate reader (Thermo Fisher Scientific, Waltham, MA, USA).

### 5.5. Statistical Analysis

All the experimental data obtained were analyzed using SPSS version 19.0 statistics software (SPSS, Chicago, IL, USA) and presented as mean ± standard deviation. Statistical differences were determined by one-way analysis of variance followed by Duncan’s multiple comparison test and were considered significant at probability values (*p*-values) of less than 0.05 (*p* < 0.05).

## Supplementary Materials

The following are available online at https://www.mdpi.com/2072-6651/12/8/497/s1, Table S1: The primer sequences of target genes and the internal reference gene (GAPDH).
