# Supplementary material for: Effects of Peptidoglycan, Lipoteichoic Acid and Lipopolysaccharide on Inflammation, Proliferation and Milk Fat Synthesis in Bovine Mammary Epithelial Cells
Source: Toxins (Basel). 2020 Aug 2;12(8):497. doi: 10.3390/toxins12080497 (PMC7472015; doi:10.3390/toxins12080497)
Supplement: Supplementary file 1 [file toxins-12-00497-s001.pdf]

Supplementary Materials: Effects of Peptidoglycan, Lipoteichoic Acid and Lipopolysaccharide on Inflammation, Proliferation and Milk Fat Synthesis in Bovine Mammary Epithelial Cells

Yongjiang Wu, Yawang Sun, Zhu Zhang, Juncai Chen and Guozhong Dong

Table S1. The primer sequences of target genes and the internal reference gene (GAPDH).

Genes	Primer sequences (5' to 3')	Product size/bp	Accession number
IL-1β	F: AGTGCCTACGCACATGTCTTC<br>R: TGCGTCACACAGAAACTCGTC	114	NM_174093.1
IL-6	F: TGCTGGTCTTCTGGAGTATC<br>R: GTGGCTGGAGTGGTTATTAG	153	NM_173923.2
IL-8	F: ATGACTTCCAAGCTGGCTGTTG<br>R: TTGATAAATTTGGGGTGGAAAG	149	NM_173925.2
TNF-α	F: CCACGTTGTAGCCGACATC<br>R: CCCTGAAGAGGACCTGTGAG	155	XM_005223596.4
FASN	F: AGGACCTCGTGAAGGCTGTGA<br>R: CCAAGGTCTGAAAGCGAGCTG	85	NM_001012669.1
ACACA	F: GATCCAGGCCATGCTAAG<br>R: CTGTTTCTCCAGCCACTC	103	XM_024979607.1
SCD	F: TCCGACCTAAGAGCCGAGAA<br>R: TGGGCAGCACTATTCACCAG	200	NM_173959.4
GAPDH	F: GGGTCATCATCTCTGCACCT<br>R: GGTCATAAGTCCCTCCACGA	176	NM_001034034.2

IL-1β, interleukin-1β; *IL-6*, interleukin-6; *IL-8*, interleukin-8; *TNF-α*, tumor necrosis factor-α; *FASN*, fatty acid synthase; *ACACA*, acetyl coenzyme-A carboxylase 1; *SCD*, stearoyl-CoA desaturase; *GAPDH*, glyceraldehyde-3-phosphate dehydrogenase.
